# Rutin Potentially Binds the Gamma Secretase Catalytic Site, Down Regulates the Notch Signaling Pathway and Reduces Sphere Formation in Colonospheres

**DOI:** 10.3390/metabo12100926

**Published:** 2022-09-29

**Authors:** Atul Kumar Singh, Mohd Shuaib, Kumari Sunita Prajapati, Shashank Kumar

**Affiliations:** Molecular Signaling & Drug Discovery Laboratory, Department of Biochemistry, Central University of Punjab, Guddha, Bathinda 151401, Punjab, India

**Keywords:** gamma secretase, notch signaling pathway, flavonoid, colon cancer, stem-like cells

## Abstract

Rutin, a natural flavonol, can modulate molecular signaling pathways and has considerable potential in cancer treatment. However, little is known about the effect of rutin on the notch signaling pathway (NSP) in cancer and cancer stem-like cells. In this study, we explored the effect of rutin on gamma secretase (GS, a putative notch signaling target) inhibition mediated NICD (Notch Intracellular Domain) production in colon cancer cells. Molecular docking, MM-GBSA, and Molecular dynamics (MD) simulation experiments were performed to check rutin’s GS catalytic site binding potential. The HCT-116 colon cancer and cancer stem-like cells (colonospheres) were utilized to validate the in silico findings. The NICD production, notch promoter assay, expression of notch target genes, and cancer stemness/self-renewal markers were studied at molecular levels. The results were compared with the *Notch-1* siRNA transfected test cells. The in silico study revealed GS catalytic site binding potential in rutin. The in vitro results showed a decreased NICD formation, an altered notch target gene (*E-cad*, *Hes-1*, and *Hey-1*) expression, and a reduction in stemness/self-renewal markers (*CD44*, *c-Myc*, *Nanog*, and *Sox2*) in test cells in a time and dose-dependent manner. In conclusion, rutin inhibits the notch signaling pathway and reduces the stemness/self-renewal property in colon cancer cells and the colonospheres by targeting gamma secretase. The clinical efficacy of rutin in combination therapy in colon cancer may be studied in the future.

## 1. Introduction

Colon cancer has been identified as one of the most common and fatal malignancies in both men and women worldwide. According to the American Cancer Society, 104,270 new cases of colon cancer will be reported in 2021. Colon cancer is a lifetime risk for 4.3 percent of men and 4.0 percent of women (including rectal cancer). Despite a reduction in CRC-related morbidity over the last decade, metastatic CRC is difficult to treat and has significant fatality rates [1,2]. The notch signaling pathway (NSP) has been shown to alter the proliferation of, and apoptosis in, colon cancer cells in recent investigations. NSP activation has been demonstrated to contribute directly to cancer cell stemness and invasion in colon cancer [3,4]. In colon cancer patients, hyperactivation of the NSP is linked to aggressiveness, high-grade tumor, and enhanced metastasis [5,6] of the tumor. Increased expression in the epithelial to mesenchymal transition (EMT)/stemness related proteins leads to EMT and stem-cell-like phenotypes in colon cancer cells after the constitutive activation of NSP [7]. Targeting the NSP for the treatment of colon cancer is gaining popularity based on data that it promotes tumorigenesis, metastasis, and drug resistance.

The notch signaling pathway (NSP) is implicated in cancer promotion, proliferation, and progression regulation. The principal players in NSP, the notch receptors (*Notch-1* to 4), are made up of an extracellular, a transmembrane, and an intracellular/cytoplasmic domain. The notch is activated when it interacts with ligands on surrounding cells and is then proteolytically cleaved (a three-step process). After the two cleavages, a membrane-bound receptor with an intracellular domain remains, which acts as a substrate for the gamma secretase (GS) complex. The notch intracellular domain (NICD) of notch 1/2/3/4 receptors is cleaved by gamma secretase (a membrane-associated enzyme), which translocates into the nucleus and triggers the transcription of target genes [8]. Compounds that inhibit GS-mediated notch receptor cleavage are a better technique for colon cancer medication discovery and are currently in clinical trials. Although notch signaling is vital for maintaining intestinal epithelial health, inappropriate activation has been linked to the development of colon cancer. Upregulation of *Notch-1* and its target gene(s) in colon cancer has been linked to tumor progression, epithelial-to-mesenchymal transition (EMT), and increased self-renewal/stemness features [8,9]. Increased treatment resistance in both colon cancer and cancer stem cells is linked to decreased overall survival, poor therapeutic outcome, and disease relapse in colon cancer patients with an upregulated notch signaling pathway. Gamma secretase inhibitors (GSIs) have been the focus of most notch signaling inhibitory research in cancer cells. The severe side effects and non-specific nature of the GSI are the main roadblocks to these inhibitors reaching the clinic. As a result, natural plant-derived molecules with minimal toxicity profiles are being investigated as *Notch-1* inhibitors [8,10].

Rutin is a natural phytochemical that belongs to the flavonol sub-group and has a double bond in the C ring, a hydroxyl group at the C3 position, and a C-O bond with the sugar moiety (O-glycosides). Although rutin is best recognized as a citrus flavone, it can also be found in various fruits, vegetables, cereals, and other foods. Buckwheat contains a high proportion of rutin (2–10 percent of plant weight). This herb has been used medicinally in the United States since the 1940s, implying that rutin’s medicinal usefulness dates back to the 1940s [11]. Rutin has been shown to target cancer cells by providing anti-inflammatory effects, suppressing pro-inflammatory secretions, altering transcription factors, and modulating cancer signaling pathways (MAPK, PI3K/Akt, epidermal growth factor (EGF), and Wnt/catenin) [12]. Rutin causes apoptosis, cell cycle arrest in the G2/M phase, and inhibits colon cancer cell proliferation, adhesion, and migration [13,14]. Furthermore, rutin can radiosensitize colon cancer cells [15]. Rutin has recently been demonstrated to regulate *Notch-1* and *Hes-1* mRNA levels in cervical cancer cells. Furthermore, rutin has been demonstrated to have modulated the signaling pathways in cancer cells and cause apoptosis [16,17]. In a recently published study, rutin was found to cause apoptosis of cervical cancer cells by downregulation of the *Notch-1* and *Hes-1* genes [18]. Rutin’s ability to modulate the notch signaling system in cancer cells has not been studied. Furthermore, the effect of rutin on cancer stem cells has not yet been described to our knowledge. Although there are just a few reports on rutin’s efficacy in normal stem cells [19,20]. Using in silico and in vitro approaches, the current study aimed to determine the ability of rutin to modulate the notch signaling pathway in colon cancer/cancer stem-like cells (colonospheres).

## 2. Materials and Methods

### 2.1. Protein, Ligand Retrieval and Preparation

The three-dimensional (3D) structure of gamma secretase enzyme complex (PDB ID: 7C9I) deposited by Yang et al. (2021) was obtained from protein data bank [21,22]. The resolution of the retrieved protein structures was 3.10 Å. Visualization of the downloaded structure was carried out by PyMol software (The PyMOL Molecular Graphics System, Version 2.0 Schrödinger, LLC). The 3D structure of DAPT and rutin was downloaded from PubChem database [23]. The DAPT and rutin were prepared for docking using the LigPrep module of the Schrödinger package 2020-1 (Schrödinger, LLC, New York, NY, USA, 2020). LigPrep corrects various structural problems of ligand molecules such as the addition of hydrogen atoms, a 2D-3D conversion, the correction of bond lengths, and energy minimization. During ligand preparation the ionization state of ligands was not changed, tautomers were not created, and ligands were allowed to retain their particular chirality. Furthermore, all atom force field charges and atom types were assigned using the optimized potential of liquid simulations of the 3 (OPLS3) force field [24,25]. The Protein Preparation Wizard of the Schrödinger program was used to prepare the proteins. Missing hydrogen atoms and side chains were added to the downloaded structure to improve its structural characteristics. Following that, a systematic, cluster-based strategy was used to optimize the hydrogen bond network of the protein structure. Restrained structure minimization was also used to allow hydrogen atoms to move freely while allowing enough heavy atom mobility to relax the strained bonds, angles, and conflicts. The OPLS3 force field was used for the energy minimization of protein structure.

### 2.2. Receptor Grid Generation and Molecular Docking

A prepared protein structure was used for grid generation for docking by using the receptor grid generation module of the Schrödinger package. The grid was generated on the basis of bound ligand in the downloaded structure. The coordinates of the generated grid were: 20 Å × 20 Å × 20 Å) X = 163.786653307, Y = 173.996831545, and Z = 148.410246426. The DAPT and rutin ligands were docked on the generated receptor grid on the receptor protein molecule by using the Glide module of the Schrödinger package. The Glide module of Schrödinger package uses the Emodel scoring function for the comparison of the ligand protein docked poses and the Glide score. Visualization, representation, and analysis of interacting residues were performed using the BIOVIA Discovery studio visualizer.

### 2.3. MM-GBSA Calculation

Prime MM-GBSA (molecular mechanics combined with the generalized Born surface area) was used to compare the binding energies of DAPT and rutin for the DAPT/rutin-bound protein complex. The protein-ligand binding free energies were calculated using the VSGB solvent model, the OPLS_2005 force field, and rotamer search algorithms [24,25,26]. The total free energy of the binding was computed using the Glide posture viewer file in the Prime MM-GBSA simulation. The MM/GBSA calculations were used to determine the relative binding affinity of a protein and its ligand. The MM-GBSA energies for both complexes were calculated by using the following equation:

ΔG_bind_ = ΔG_complex_ − (G_receptor_ + G_ligand_) (1)

where ΔG_bind_ represents binding the free energy of the receptor-ligand complex, which can be decomposed into binding the free energy of the complex (ΔG_complex_), the receptor (G_receptor_), and the ligand (G_ligand_). The free energy ΔG can be obtained by the following equations:

ΔG = ΔE_gas_ + ΔG_sol_ − TΔS_gas_ (2)

ΔE_gas_ = ΔE_elec_ + ΔE_vdw_ (3)

ΔG_sol_ = ΔG_gb_ + ΔG_surf_ (4)

In above equations ΔE_gas_ represents the molecular mechanics energy in the gas phase, which can be calculated as the sum of electrostatic interactions (ΔE_elec_) and Van der Waals interactions (ΔE_vdw_) while TΔS_gas_ represents the entropy terms. The solvation energy term was calculated as the sum of polar solvation energy (ΔG_gb_) and non-polar solvation energy (ΔG_surf_) [27,28]. The MM/GBSA binding energies are estimates of binding free energies, therefore, a lower number indicates greater binding [29].

### 2.4. Molecular Dynamics Simulation

For the preparation of the systems for molecular dynamics (MD) studies, the unbound protein and the protein in complex with DAPT and rutin were submitted to the CHARMM-GUI server [30]. Ligand parameters and topology for the DAPT and rutin were generated using the ParamChem (www.paramchem.org) service available at the CHARMM-GUI server [31]. In the first step, the orientation of the protein with respect to the membrane was determined using the PPM server [32]. The PPM server uses 3D architectures to estimate the rotational and translational locations of transmembrane and peripheral proteins. In the second step, the membrane components were generated using the Membrane Builder module of CHARMM-GUI server [33]. The membrane system was generated using 100 Dipalmitoylphosphatidylcholine (DPPC) molecules in both the upper and lower leaflet of the membrane. In the third step, unbound protein and protein in complex with DAPT and rutin were inserted into the membrane using the replacement method. The rectangular box type was selected for the preparation of the system. Neutralizing ions were added to the system by using the Monte Carlo ion placing method. In the fourth step, the water box was generated to fully solvate the generated membrane protein system. The TIP3P water model was used for the solvation of the system. In the fifth step, all the generated components were assembled to form simulation systems. The CHARMM36 force field was applied for the simulation of generated systems [34]. Furthermore, input files were generated for the GROMACS MD simulation package [35]. Energy minimization of all generated systems was performed using the 5000 steps of the steepest descent method [36]. Next, all the generated systems were equilibrated by using the six-step equilibration protocol of the CHARMM-GUI. During equilibration temperature was maintained at 300 K by applying the Berendsen thermostat method [37]. However, for the production run the temperature was maintained at 300 °C by applying the Nose–Hoover thermostat [38]. Similarly pressure of the generated systems was maintained at 1 bar by using a Parrinello–Rahman barostat [39]. Long range interactions were handled using the Linear constraint solver (LINCS) algorithm [40]. A Verlet cutoff scheme was used for the production run. Finally, all the systems were simulated for 150 nanoseconds (ns) and trajectories were recorded at an interval of 2 picoseconds (ps). The GROMACS 2020.4 package was used to conduct MD simulation of all systems [41].

### 2.5. Trajectory Analysis

Various analytic modules included in the GROMACS package were used to perform trajectory analysis. The *gmx_rms* and *gmx_rmsf* modules were used to determine the root mean square deviation (RSMD) and the root mean square fluctuation (RMSF). Radius of gyration (Rg) and solvent accessible surface area (SASA) were determined using the *gmx_gyrate* and *gmx_sasa* modules, respectively. The hydrogen bonding network and the secondary structure were determined using the *gmx hbond* and *gmx do_dssp* modules of GROMACS 2020.4. The visualization of trajectories was performed using the Visual Molecular Dynamics (VMD) software and graphical representations were prepared using the Grace software (https://plasma-gate.weizmann.ac.il/Grace/) [42].

### 2.6. Principal Component Analysis and Free Energy Landscape

Principal component analysis (PCA) is a widely applied technique to identify patterns in high dimensional data [43]. PCA is often used as a technique in exploratory data analysis to illustrate the internal data structure so that it can explain the variance in the data. When applied to a group of experimental structures or MD trajectories it can explain the concerted atomic displacement and can highlight the major conformational changes between structures. PCA is applied in the analysis of a set of experimental structures or MD trajectories for the purposes of explaining the concerted atomic displacement and highlighting the major conformational changes in the structure. These concerted atomic displacements and conformational changes are required for the functioning of protein structures. Mathematically, the principal components of the MD simulation trajectory are derived by the diagonalization of the data covariance matrix C:

C = V*Λ*V^T^ (5)

This provides the diagonal matrix *Λ,* which possesses the eigenvalues as diagonal entry and matrix V, which possess the corresponding eigenvectors [44]. The GROMACS analysis tools *gmx_covar* and *gmx_anaeig* were used for the principal component analysis.

The free energy landscapes (FEL) were calculated using the *gmx_sham* tool of GROMACS [45,46]. First two principal components were used to calculate the FEL using the following equation:

ΔG (PC1, PC2) = −K_B_TlnP (PC1, PC2) (6)

where PC1 and PC2 symbolize the reaction coordinates, K_B_ represents the Boltzmann constant, and P (PC1, PC2) represent the probability distribution of the system. FEL was plotted using OriginLab software.

### 2.7. Reagents

Dulbecco’s Modified Eagle Medium (DMEM), fetal bovine serum (FBS), trypsin, penicillin, streptomycin, RNAase A, and trizol were from Thermo Fisher Scientific, (Waltham, MA, USA). The MTT (3-(4,5-dimethylthiazol-2-yl)-2,5-diphenyl tetrazolium bromide) was obtained from Sigma-Aldrich, St. Louis, MO, USA. Biorad, Hercules, CA, USA, provided the iScript^TM^ cDNA synthesis kit and the SYBR^®^ green master mix. The *Hes-1* (PA528802), *c-Myc* (700648), and NICD (ab8925), antibodies were procured from Invitrogen-Thermo Fisher Scientific, Waltham, MA, USA and Abcam, Cambridge, UK. The β-actin secondary antibody (4970S) was purchased from Cell Signaling Technology, Danvers, MA, USA. Rutin (Purity: 97.09%) (S2350) was purchased from Selleckchem.

### 2.8. Cell Culture and MTT Assay

Human colon cancer cells HCT-116 were purchased from the National Centre for Cell Sciences, Pune, India. The cells were cultured in DMEM nutrient media supplemented with 10% (*v/v*) heat inactivated FBS, L-glutamine (2 mM), penicillin (100 U/mL), and streptomycin (100 μg/mL). The cells were maintained at 37 °C under a humid environment and 5% CO_2_ in an incubator. An MTT assay was performed to check the efficacy of rutin in HCT-116 cells and to calculate the inhibitory IC_30_ and IC_50_ concentrations as per the protocol and calculation described elsewhere [47,48]. Briefly, the colon cancer cells were seeded in a 96-well plate at a density of 1 × 10^4^ cells/well in triplicate. The cells were treated with rutin at a concentration range of 10–500 μM for 24 h. After incubation, the wells were given a 10 μL MTT solution (5 mg/mL) and incubated at room temperature for 4 h. The formed crystals were dissolved by adding DMSO (100 μL) to the wells followed by an optical density measurement (at 590 nm) using a microplate reader (BioTek Instruments, Inc., Winooski, VT, USA).

### 2.9. Silencing of Notch-1 by siRNA

The formation of the Notch Intracellular Domain (NICD), due to the cleavage of the *Notch*-1 receptor by the gamma secretase complex in the HCT-116 cancer cells and the derived colonospheres, was blocked by using synthetic *Notch-1* siRNA as mentioned earlier [49]. Briefly, the cells were transfected with 30 nM of synthetic siRNA duplexes (sense 5-CAACAACAAUGAGUGUGAAtt, antisense 5-UUCACACUCAUUGUUGUUGAT; Ambion, Life Technologies) for 24 h to silence *Notch-1* expression using Dharmafect Transfection Reagent (Life Technologies). The transfected cells were then used for further experiments. For the negative control experiments, cells were transfected with 30 nM of scrambled control sense siRNA.

### 2.10. Colonosphere Formation and Phytochemical Treatment

Colonosphere (colon cancer stem-like cells) formation mimics the colon cancer stem cells’ properties and has been used to study the molecular mechanism of the anti-colon cancer stem cell property of lead molecules [50]. The colon cancer cell line (HCT-116) was utilized to form colonospheres. A total of 2 × 10^4^ cells/well were seeded in 6-well ultra-low-attachment surface plates (Corning, New York, NY, USA). The cells were cultured in serum-free DMEM-F12 (1:1, *v/v*) supplemented with the necessary ingredients described in previous studies [48]. After that, the cells were incubated in a humid environment (37 °C, 5% CO_2_) for five days to allow the formation of colonospheres [51]. To study the effect of rutin on colonosphere formation, the cells were pre-treated at IC_30_ and IC_50_ concentrations (obtained in the MTT assay in HCT-116 cells). Similarly, the vehicle-treated HCT-116 cells were allowed to form colonospheres and considered as the control group. For the experiments, rutin was dissolved in DMSO, thus an equal amount of DMSO was added to vehicle-treated colonosphere group. Following this, the *Notch-1* siRNA and siRNA-control transfected HCT-116 cells were incubated for five days and allowed to form colonospheres. After the incubation the colonospheres from the test groups were photographed using microscopy. The images were assessed through Image J software.

### 2.11. Notch Promoter Activity

The notch promoter activity was measured using a dual-luciferase assay. We used the pGL4[luc2P/RBP-JK-RE/Hygro] vector with the NICD binding site (notch promoter) linked to the luciferase gene in this test. The internal control was the p[Rluc-Neo/SV40] vector (Renilla). In a 96-well plate, HCT-116 cells (4 × 10^4^ cells/well) were plated. The pGL4[luc2P/RBP-JK-RE/Hygro] and p[Rluc-Neo/SV40] vectors were transfected into the cells using the Lipofectamine 3000 transfection reagent (Thermo Fisher Scientific, Waltham, MA, USA) according to the manufacturer’s instructions. The cells were treated at a rutin IC_50_ concentration for 48 h and 72 h and the cells were incubated at 37 °C. On a GloMax 20/20 Luminometer, the Luciferase activity was evaluated using the Dual-Luciferase Reporter Assay Kit (Promega Corporation, Fitchburg, WI, USA).

### 2.12. RNA Isolation, cDNA Synthesis, and Quantitative RT-PCR Analysis

HCT-116 cells were seeded in 6-well plate at a density of one million cells per well followed by overnight incubation. Treatment was carried out with the IC_50_ concentration of rutin for 48, and 72 h. Similarly, both the vehicle-treated and rutin pre-treated (at the IC_30_ and IC_50_) HCT-116 derived colonospheres were utilized for qRT-PCR experiments. The *Notch-1* siRNA and siRNA-control transfected HCT-116 cells and colonospheres were also utilized for the qRT-PCR experiments. RNA was isolated from the HCT-116 cells and colonosphere test groups using TRIzol reagent (Thermo Scientific Fisher, Waltham, MA USA) following the manufacturer’s guidelines. The quality of total RNA was analyzed using a Nanodrop 2000 spectrophotometer. For reverse transcription to cDNA, samples having an absorbance range of 1.90–2.0 at a 260/280 nm ratio were used. The iScript^TM^ cDNA Synthesis Kit was used to reverse-transcribe RNA (1 µg) into cDNA (BIORAD). The SYBR Green PCR Master Mix and the synthesized cDNA were utilized in a qPCR process (BIORAD). To synthesize cDNA the sequence of primers for the target genes (*GAPDH*, *Hes-1*, *Hey-1*, *E-cad*, *Nanog*, *Sox2*, *c-Myc,* and *CD44*) were tabulated as shown in the Supplementary Table S1. The reaction was carried out in a Veriti^®^ 96-well fast thermal cycler (Applied Biosystems) using the protocol start (95 °C, 5 min), denaturation (95 °C, 30 s), annealing (55 °C, 45 s), and elongation at 72 °C for 45 s [9,51]. GAPDH was used for normalization.

### 2.13. Western Blotting

The HCT-116 cells (5 × 10^4^) were treated with the IC_30_ and IC_50_ concentrations and incubated for five days to allow the sphere formation; and the similar set of vehicle-treated cells were allowed to form spheroids as per the methodology given in Section 2.10. After the incubation, the colonospheres were harvested and the protein concentration was measured using the bicinchoninic acid method. For the Western blotting experiment the obtained protein sample was resolved on SDS-PAGE followed by transfer to the PVDF membrane. The PVDF membrane was blocked, washed, and incubated at 4 °C for 12 h with human specific primary antibodies for *c-Myc* (1:1000× dilution, Catalog: 700648, Invitrogen, Thermo Fisher Scientific, USA), Anti-activated *Notch-1* (1:250× dilution, Catalog: ab8925, Abcam, Cambridge, UK)), *Hes-1* (1:500× dilution, Catalog: PA528802, Invitrogen, Thermo Fisher Scientific, USA), and (β-actin with 1:1000× dilution, Catalog: MA532540, Invitrogen, Thermo Fisher Scientific, USA). The membrane was then treated for 2 h at 37 °C with a secondary antibody that was labeled with horseradish peroxidase. Β-actin was used as an internal control. The blots were then visualized by an enhanced chemiluminescence (ECL) system (Thermo Fisher Scientific). The images were recorded using image lab software 6.0.1 Bio-Rad [52].

### 2.14. Statistical Analysis

All of the studies were done in triplicate, and the findings were given as means ± SD. Statistical analysis was done using a one-way ANOVA test. GraphPad Prism was used to conduct the statistical analysis.

## 3. Results and Discussion

The process of creating new inhibitor compounds is time-consuming and difficult. Drug repurposing is a valuable strategy in drug development that entails testing established drug molecules for inhibitory potential against new drug targets. Furthermore, compared to de novo drug discovery, medication repurposing is more cost-effective and less risky because the pharmacokinetic and pharmacodynamic properties of existing pharmacological molecules are already known. The medicinal molecule must, however, successfully engage the target protein to function as an inhibitor [53,54]. With these facts in mind, we used a typical in silico approach to investigate the gamma secretase catalytic site binding potential of rutin.

### 3.1. Rutin Binds Efficiently to the Gamma Secretase Catalytic Site

**Figure 1:**
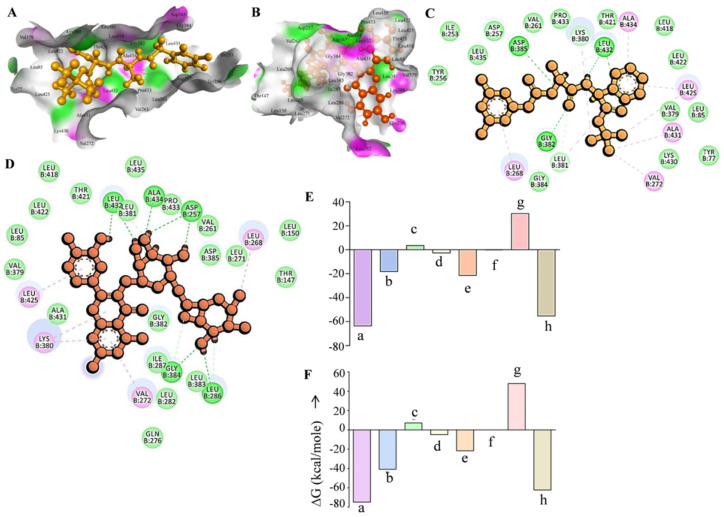
Docking and MM-GBSA analysis of rutin and DAPT with the gamma secretase catalytic site. (**A**) Interaction of DAPT with gamma secretase active site represented with hydrogen bond surface of receptor. (**B**) Interaction of rutin with gamma secretase active site represented with hydrogen bond surface of receptor. (**C**) Residual interaction of DAPT with the gamma secretase active site. (**D**) Residual interaction of rutin with the gamma secretase active site. (**E**) Plot of MM-GBSA binding energy estimation of the DAPT-gamma secretase complex represented with component energy terms. (**F**) Plot of MM-GBSA binding energy estimation of the rutin-gamma secretase complex represented with component energy terms. a—Total binding energy, b—Coulombic interaction energy, c—Covalent interaction energy, d—Hydrogen bond interaction energy, e—Lipophilic interaction energy, f—Pi-pi packing interaction energy, g—Generalized Born electrostatic solvation energy, and h—Van der Waal’s interaction energy.

Molecular docking experiments are the most extensively utilized method for determining a medicinal molecule’s binding affinity to a certain target [55,56]. Thus, we tested the binding efficiency of rutin against the gamma secretase catalytic subunit (GSCS) using molecular docking. The known gamma secretase inhibitor DAPT was used for molecular docking investigations as the reference molecule. Molecular docking experiments were carried out on a grid derived from the PDB structure of bound gamma secretase inhibitor. In comparison to the typical gamma secretase inhibitor DAPT, rutin showed outstanding binding efficacy with the GSCS in our docking tests (Figure 1). With the GSCS, rutin had a dock score of −14.77, while the typical GSI DAPT had a dock value of −9.2. Five hydrogen bonds with amino acid residues Asp257, Leu286, Gly384, Leu432, and Ala434 allowed rutin to attach to the gamma secretase catalytic subunit, whereas three hydrogen bonds with amino acid residues Gly382, Asp385, and Leu432 allowed DAPT to bind to the catalytic subunit (Figure 1A–D). Furthermore, the gamma secretase catalytic unit’s Lys380, Ala431, Leu425, Val379, Leu85, Leu422, Leu418, Thr421, Leu381, Leu435, Pro433, Val261, Asp385, Leu271, Leu268, Leu150, Thr147, Gln276, Val272, Ile287, Leu282, Gly382, and Leu383 amino acids showed hydrophobic interaction with rutin. On the other hand, the gamma secretase catalytic unit’s Tyr256, Ile253, Leu435, Asp257, Val261, Pro433, Lys380, Thr421, Ala434, Leu418, Leu422, Leu425, Val379, Leu85, Ala431, Lys430, Tyr77, Val272, Leu381, Gly384, and Leu268 amino acid residues interacted with DAPT (Figure 1A–D). Leu432 was a frequent amino acid residue establishing hydrogen bonds with gamma secretase in both complexes. Additionally, Lys380, Ala431, Leu425, Val379, Leu85, Leu422, Leu418, Thr421, Leu381, Leu435, Pro433, Val261, Leu268, and Val272 were found in both complexes to make hydrophobic interactions with the GSCS (Figure 1A–D). The Asp257 and Asp385 residues are required for the gamma secretase enzyme complex to function [57,58]. In our study we found that rutin formed a hydrogen bond with Asp257 and a hydrophobic interaction with Asp385, whereas DAPT had a hydrogen bond interaction with Asp385 and a hydrophobic interaction with Asp285. Another common gamma secretase inhibitor, L-685,458, is said to make hydrogen bonds with the catalytic subunit’s Lys380, Gly382, Gly384, Lys432, Asp257, and Asp285 amino acid residues [21]. Asp257, Gly384, and Leu432 were among the amino acid residues forming hydrogen bonds with the rutin. Semagacestat and avagacestat, two more common gamma secretase inhibitors, have also been discovered to bind on the same binding site of the gamma secretase catalytic subunit. Yang et al. (2021) proposed that the Presenilin component of the gamma secretase enzyme complex functions as both a substrate and an inhibitor binding site [21]. Based on these findings, it may be concluded that rutin binds to the catalytic/inhibitor binding site of the gamma secretase subunit.

We used the MM-GBSA approach to determine the binding energy of the rutin-gamma secretase complex (RGSC) and the DAPT-gamma secretase complex (DGSC) to gain further insight into their comparative binding efficacy to GSCS. The MM-GBSA method is a popular method for calculating the binding energies of protein-ligand complexes. In comparison to DAPT, MM-GBSA binding energy calculation demonstrated that rutin has a greater binding efficiency than the GSCS. The binding energy of the RGSC was −74.84 kJ/mole, while the binding energy of the DGSC was −63.62 kJ/mole (Figure 1E,F). The overall binding energy of both complexes was dominated by Coulombic interaction energy, lipophilic interaction energy, and Van der Waal’s interaction energy. The Van der Waal’s interaction energy and the lipophilic interaction energy were identical in both complexes, but the Coulombic interaction energy was much higher in the RGSC than in the DGSC, resulting in a considerable difference in total binding energy. Thus, based on dock score and MM-GBSA binding energy estimation, it can be concluded that rutin binds to the gamma secretase catalytic subunit more efficiently than DAPT.

### 3.2. Rutin Forms Stable and an Energetically Favorable Complex with a Gamma Secretase Catalytic Subunit

**Figure 2:**
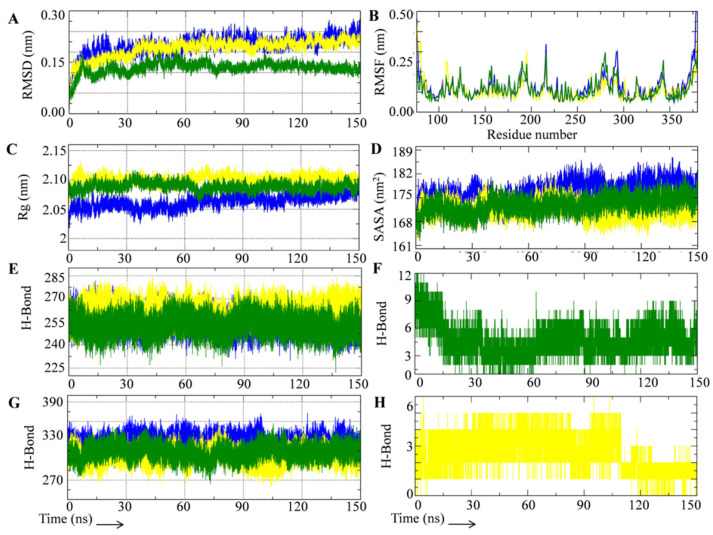
MD simulation trajectory analysis of test systems during 150 ns simulation period. (**A**) RMSD plot of UGSC (blue), DGSC (yellow) and RGSC (green). (**B**) RMSF plot of UGSC (blue), DGSC (yellow) and RGSC (green). (**C**) Rg plot of UGSC (blue), DGSC (yellow) and RGSC (green). (**D**) SASA plot of UGSC (blue), DGSC (yellow) and RGSC (green). (**E**) Average number of intramolecular hydrogen bonds formed in UGSC (blue), DGSC (yellow) and RGSC (green). (**F**) Average number of hydrogen bonds formed between rutin and GS. (**G**) Average number of hydrogen bonds formed between GS and surrounding solvent in UGSC (blue), DGSC (yellow) and RGSC (green). (**H**) Average number of hydrogen bonds formed between DAPT and GS.

MD simulations are a common method for determining the stability and interaction pattern of protein-ligand complexes [59,60,61]. The GROMACS MD simulation program was used to simulate the unbound gamma secretase catalytic subunit (UGSC), as well as DGSC and RGSC. In traditional MD simulations, atoms and molecules are permitted to physically move for a brief amount of time, with the applied force field governing the forces between them. To establish the dependability of MD simulation of all the three systems, quality check parameters such as temperature, pressure, potential, and kinetic energy were assessed. Throughout the 150 ns MD simulation period, each parameter displayed a stable and equilibrated pattern (Supplementary Figure S1). We also looked at the structural stability and interaction pattern of DAPT and rutin with the gamma secretase catalytic subunit by looking at several structural order parameters like root mean square deviation (RMSD), root mean square fluctuation (RMSF), radius of gyration (Rg), solvent assessable surface area (SASA), and hydrogen bond formation, as shown in Figure 2. The structural deviation of the backbone in the unbound condition and after the binding of DAPT and rutin was determined using RMSD analysis of all three simulated systems. Starting at 0.1 nm, all three systems showed an increase in RMSD, but by 10 ns, they had equilibrated. There is no substantial increase in RMSD after 10 ns in all three systems until the 150 ns simulation period. Among the three simulated systems, UGSC had the highest RMSD, followed by DGSC and RGSC. In comparison to the unbound and DAPT-bound GSCS, rutin-bound GSCS had a significantly lower RMSD. The average RMSD values of all three systems with standard deviations are summarized in Supplementary Table S1. RGSC had an average RMSD of 0.16 nm, much lower than the UGSC (0.21 nm) and the DGSC (0.20 nm). Figure 2A shows that the rutin-bound gamma secretase catalytic subunit showed an equilibrated RMSD pattern, indicating that both entities formed a stable complex. Furthermore, the RGSC’s stable RMSD pattern suggests that rutin was spatially well-occupied and stabilized by molecular contacts with the gamma secretase catalytic subunit’s binding pocket. The RMSF calculation was used to determine the positional variability of amino acids relative to their average mean positions. The positional changes of certain amino acids and structural domains of biomolecular complexes are revealed by RMSF analysis. In the case of proteins, larger fluctuations are seen in the terminal and looped region. Lower fluctuations in protein structure imply that -helices and -sheets have a stable secondary structure. The RMSF plots of all three complexes show that ligand attachment to the gamma secretase catalytic subunit alters the RMSF pattern (Figure 2B). Although DAPT binding reduced some of the biggest peaks in the UGSC RMSF plot, it also caused changes in other areas. Similarly, rutin binding reduced residual fluctuations in some gamma secretase catalytic subunit locations, but increased residual fluctuations in others. UGSC had the higher average RMSF value (0.12 nm), followed by RGSC (0.11 nm), and DGSC (0.10 nm) (Supplementary Table S1).

The compactness of the protein structure was examined using Rg, which helps to understand the simulated systems’ comparative structural stability. The folding behavior of protein complexes can be predicted using the Rg analysis. A well-folded protein’s Rg plot will remain generally stable, while alterations in the Rg plot reflect protein structure unfolding. Figure 2C shows Rg plots for all three simulated systems, and Supplementary Table S1 lists average Rg values. The Rg plots of all three systems imply that the gamma secretase catalytic subunit has a consistent folding pattern. Lower Rg values in MD simulations suggest that the simulated proteins are compact. In our study, the UGSC had the lowest average Rg value (2.06 nm), followed by the RGSC (2.088), and the DGSC (2.10 nm). However, while the Rg plot of the UGSC increased somewhat over 150 ns, the Rg plots of the RGSC and DGSC remained steady, implying that rutin and DAPT binding keeps the gamma secretase catalytic subunit in a similar folding state without major changes in compactness. At the beginning of the MD simulation, the RGSC had a higher Rg value than the UGSC, but after 50 ns, it was closer to the UGSC. Furthermore, the average Rg value of the RGSC was lower than that of the DGSC, implying that rutin binding resulted in a more compact complex formation than DAPT. The slight rise in Rg values reported in both the RGSC and DGSC could be attributable to a spatial rearrangement in the gamma secretase catalytic subunit’s ligand binding site. SASA is another key attribute to investigate in MD simulation experiments to better understand biomolecular complex conformational stability. The solvent that surrounds proteins is important for maintaining its folding behavior, and the protein-ligand interaction process and stability. SASA was calculated for all three complexes and plotted in Figure 2D, with average values summarized in Supplementary Table S1. Throughout the 150 ns MD simulation period, SASA plots of all three complexes were fairly equilibrated. The UGSC had the highest average SASA value (176 nm^2^), followed by RGSC (172 nm^2^) and DGSC (171 nm^2^). In comparison to the RGSC and DGSC, the UGSC had a greater area to interact with the solvent, according to the SASA study.

Hydrogen bonds are critical for protein folding and provide structural stiffness. The intra and inter-hydrogen bond formation events in all the three simulated systems were studied, and the results are shown in Figure 2E–H. Internal structural rearrangement in proteins is indicated by changes in the pattern of intramolecular hydrogen bonds during MD simulation. The structure of the GSCS in the unbound and ligand-bound states was investigated using intramolecular hydrogen bonding in all three simulated systems. The systems displayed a fairly steady intramolecular hydrogen bond plot (Figure 2E). The higher intramolecular hydrogen bonding was found in the DGSC, followed by the UGSC and RGSC. Supplementary Table S1 shows the average intramolecular hydrogen bond formation values during the course of a 150 ns MD simulation. We looked at hydrogen bond formation between the surrounding solvent and the gamma secretase catalytic subunit in unbound and ligand-bound states for better understanding of the interaction. Figure 2G shows plots of hydrogen bond formation between the surrounding solvent and the three simulated solvents, with average values in Supplementary Table S1. The binding of ligands (rutin/DAPT) decreased the hydrogen bond interaction in the GSCS with the surrounding solvent (Figure 2G). In comparison to the RGSC and DGSC, analysis of hydrogen bond formation between the solvent and the simulated systems suggests that the UGSC had higher contact with the surrounding solvent. The creation of hydrogen bonds between the protein and the ligand is a direct indicator of potential binding. To determine the relative binding strength, time evolution graphs of hydrogen bond formation between the gamma secretase catalytic subunit and the rutin/DAPT were studied. The hydrogen bond formation analysis employed a 3.5 cutoff distance and a 30° cutoff angle. During the MD simulation, the results of the hydrogen bonding between the protein and ligand revealed that the binding of rutin with GSCS had a considerably higher interaction than DAPT (Figure 2F,H). Initially, rutin had a maximum occupancy of 12 hydrogen bonds, which decreased to seven at 15 ns and six at 45 ns, but hydrogen bonding interaction increased at 60 ns and remained stable until the end of the MD simulation period. DAPT, on the other hand, had a maximum occupancy of five hydrogen bonds, which declined to two at 110 ns. In comparison to the known inhibitor DAPT, the analysis of the hydrogen bond formation between the protein and ligand revealed that rutin had a much higher binding strength with the gamma secretase catalytic subunit.

**Figure 3:**
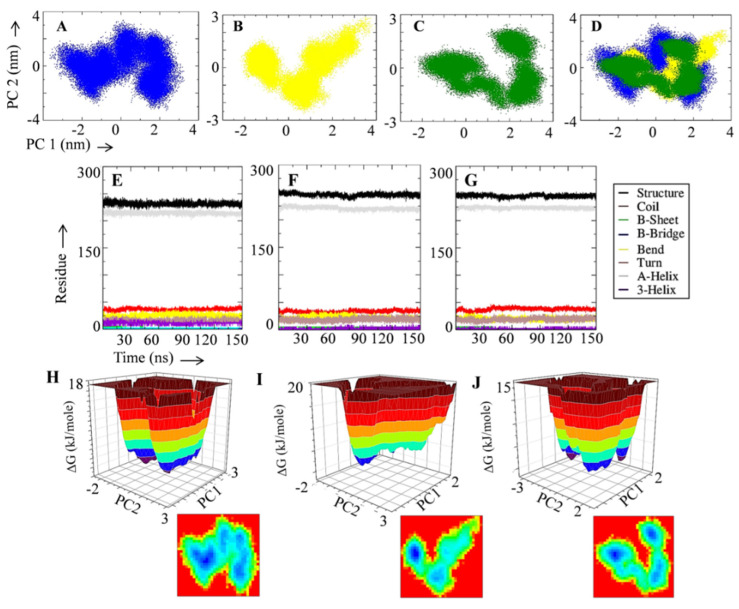
Principal component analysis, secondary structure and free energy landscape of test system during 150 ns simulation period. (**A**) PCA plot of UGSC. (**B**) PCA plot of DGSC. (**C**) PCA plot of RGSC. (**D**) Superimposed PCA plot of UGSC, DGSC, and RGSC. (**E**) Evolution of secondary structure of UGSC during 150 ns MD simulation. (**F**) Evolution of secondary structure of DAPT-bound GS during 150 ns MD simulation. (**G**) Evolution of secondary structure of rutin-bound GS during 150 ns MD simulation. (**H**) 2D and 3D free energy landscape of UGSC. (**I**) 2D and 3D free energy landscape of DGSC. (**J**) 2D and 3D free energy landscape of RGSC.

PCA is a popular dimensionality reduction method for extracting important observations from large datasets [42,62]. The mobility modes of the UGSC, DGSC, and RGSC complexes along two main components were studied using PCA. Figure 3A–D shows PCA plots of the UGSC, DGSC, and RGSC complexes alone and in superimposed states. The order of restricted motion along the principal components (PC2 and PC1) was found in the sequence of the RGSC, DGSC, and UGSC complexes (Figure 3A–D). UGSC has a wider range of motion modes along both primary components. The binding of a ligand (DAPT/rutin) restricted the respective complex’s overall motion, indicating a stable complex formation. The influence of ligand binding (DAPT, rutin) on the structural organization of protein molecules is studied via secondary structure analysis. Figure 3E–G depicts the secondary structure analyses of the UGSC, DGSC, and RGSC complexes. In comparison to UGSC, the ligand-bound complexes had a higher number of residues in the structured region and this indicates that the protein is not deformed and the binding of rutin stabilized its secondary structure. The UGSC, DGSC, and RGSC system free energy landscape analyses for 150 ns MD simulation were undertaken to study the low energy conformations on the test systems. The first and second main components generated from the PCA were used to plot the 3D and 2D free energy graphs. Figure 3H–J depicts the free energy landscapes of the UGSC, DGSC, and RGSC system. The energetically favorable conformations are represented by blue dots in the free energy landscape, whereas the energetically unfavorable conformations are represented by red spots. The free energy landscape of the RGSC complex showed more intense blue color dots than the UGSC complex, and the DGSC systems indicate that rutin binding to the GS-catalytic site reduced the energy of the system.

### 3.3. Rutin Treatment Decreases Notch Promoter Activity and Alters Notch Target Gene Expression in HCT-116 Cells

**Figure 4:**
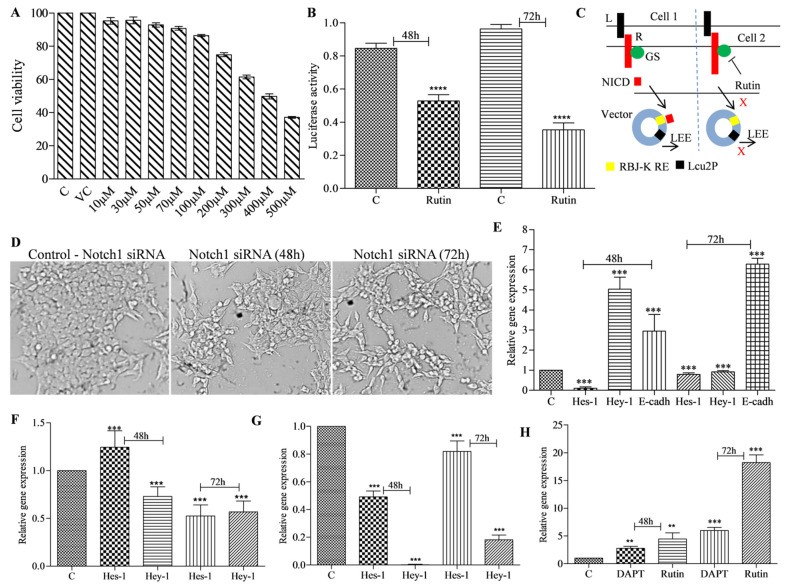
Cytotoxic potential, NICD production inhibition, and notch target gene expression modulation potential of rutin in HCT-116 cells. (**A**) Effect of rutin on cell viability of HCT-116 cells in 24 h treatment at 10–500 µM concentrations. (**B**) Notch promoter activity modulation potential of rutin at IC_50_ concentration in 48 h and 72 h treatment in notch promoter vector transfected HCT-116 cells. (**C**) Mechanism of gamma secretase mediated NICD formation (absence or presence of rutin) and thereby transcription of notch promoter region having luciferase enzyme gene. (**D**) Effect of *Notch-1* siRNA mediated silencing of *Notch-1* in HCT-116 cells survival in 48 h and 72 h transfection. (**E**) mRNA expression of *Hes-1*, *Hey-1*, and *E-cad* in *Notch-1* siRNA transfected HCT-116 cells in 48 h and 72 h treatments. (**F**) mRNA expression of *Hes-1* and *Hey-1* after 48 h and 72 h rutin treatment in HCT-116 cells at an IC_50_ concentration. (**G**) mRNA expression of *Hes-1* and *Hey-1* in DAPT (25 µM) treated HCT-116 cells in 48 h and 72 h treatments. (**H**) mRNA expression of *E-cad* after 48 h and 72 h DAPT (25 µM) and rutin (IC_50_ concentration) treatment in HCT-116 cells. Experiments were performed in triplicate and represented by mean ± SEM (standard error mean). ** (*p* < 0.01), *** (*p* < 0.001), **** (*p* < 0.0001).

Cancer cells exhibit uncontrolled cell proliferation. The antiproliferative action of rutin was tested by treating HCT-116 colon cancer cells with varying doses of rutin for 24 h and measuring cell growth. In HCT-116 cells, rutin administration inhibited cell growth in a dose-dependent manner (Figure 4A). The IC_50_ (393 µM) and IC_30_ (168 µM) concentrations were calculated using the methodology described in Section 2.8. Further experiments were carried out using the IC_50_ and/or IC_30_ concentrations. Notch receptors lose their ectodomains after ligand engagement, followed by the release of the notch intracellular domain (NICD) via gamma secretase-mediated receptor cleavage. The NICD translocates to the nucleus and interacts with the notch promoter repressor complex. The interactions activate the promoter and stimulate the expression of target genes. Inhibition of NICD production by targeting GS protein is one of the important mechanisms to downregulate the NSP in cancer cells. Driving fluorescent or bioluminescent reporters in experimental cells using a notch-responsive promoter is a sensitive method for screening the NSP activity. To determine whether rutin has inhibitory effects on gamma secretase activity, we measured the notch promoter, luciferase, activity to see if NICD is generated in rutin treated HCT-116 cells as per the methodology discussed in Section 2.11. Inhibitors of the GS enzyme stop the formation of NICD, which reduces downstream transcription of notch-targeted genes and thus inhibits the pathway. The transfected cells that had not been treated in our experiment displayed luciferase gene transcription. In transfected-HCT-116 cells treated with the rutin IC_50_ concentration, luciferase gene transcription was drastically reduced in a time-dependent manner (Figure 4B). Thus, the promoter assay showed that rutin has the potential to inhibit gamma secretase and thereby stop the NICD formation in HCT-116 cells. Previously our research group showed that phytochemicals possess the potential to inhibit NSP at the promoter level [9]. We have now studied the effect of rutin treatment on the expression profile of notch target genes in HCT-116 cells. Previously we showed that GS-mediated NICD production and its nuclear localization is crucial for the expression of notch target genes. Therefore, we used *Notch-1* receptor specific siRNA to silence the *Notch-1* in HCT-116 cells. *Notch-1* siRNA effectively silenced the *Notch-1* expression in test cells compared to scrambled (non-targeted) siRNA and caused decreased cell viability (Figure 4D). The NICD level in *Notch-1* siRNA transfected cells (at 48 h and 72 h) and the respective negative control HCT-116 cells are given in Supplementary Figure S2. The qRT-PCR analysis of the notch target genes in the *Notch-1* siRNA transfected cells showed decreased *Hes-1* and *Hey-1* and increased *E-cad* gene expression (Figure 4E). Similarly, treatment with rutin at the IC_50_ concentration significantly decreased the *Hes-1* and *Hey-1* mRNA levels (Figure 4F). Moreover, treatment with standard GSI (DAPT) at 25 µM concentration significantly decreased the *Hes-1* and *Hey-1* mRNA levels (Figure 4G). The results showed that DAPT and rutin treatment also increased the expression of *E-cad* mRNA levels in a time-dependent manner in the 48 h and 72 h treatments (Figure 4H). Studies conducted by other investigators revealed that rutin lowers the expression of *Hes-1* and *Notch-1* genes in cervical cancer cells [18]. Furthermore, rutin also induced apoptosis of cervical cancer cells by interacting with Jab1 [17]. It has been reported that notch signaling is involved in the regulation of cell-to-cell connection and cell motility which are important for the EMT of cancer cells. In the present study, we found that rutin treatment resulted in the increased expression of *E-cad* which indicates the EMT reduction capability of this phytochemical in cancer cells.

### 3.4. Rutin Decreases Colonosphere Formation by Downregulating Notch Signaling Pathway

**Figure 5:**
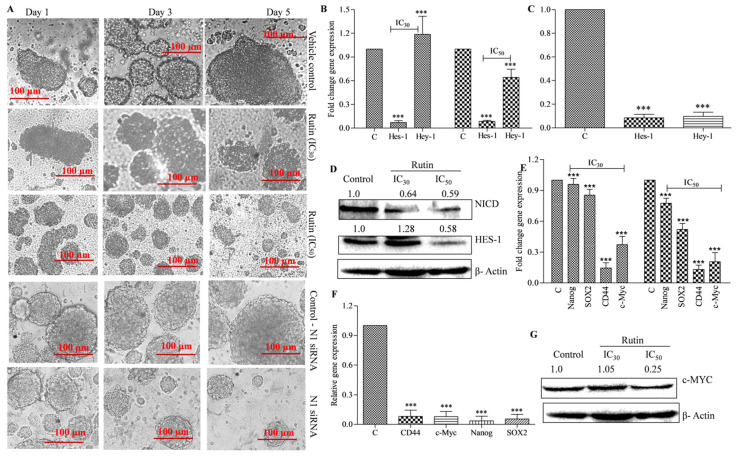
Effect of rutin on gamma secretase mediated Notch signaling pathway inhibition in colon cancer sphere forming cells. (**A**) Reduction in colony formation ability of HCT-116 cells at IC_30_ and IC_50_ concentrations of rutin/*Notch-1* siRNA treatment. (**B**) mRNA expression of *Hes-1* and *Hey-1* at IC_30_ and IC_50_ concentrations of rutin in HTC-116 derived colonospheres (*Hey-1* decrease was not observed with Rutin IC_30_). (**C**) mRNA expression of *Hes-1* and *Hey-1* in *Notch-1* siRNA treated HCT-116 derived colonospheres. (**D**) Expression of NICD and *Hes-1* in derived colonospheres at rutin IC_30_ and IC_50_ concentrations at protein level. (**E**) mRNA expression of *Nanog, Sox2, CD44,* and *c-Myc* in HCT-116 derived colonospheres at rutin IC_30_ and IC_50_ concentrations. (**F**) mRNA expression of *Nanog, Sox2*, *CD44 c-Myc* in *Notch-1* siRNA treated HCT-116 derived colonospheres. (**G**) Protein expression of *c-Myc* in HCT-116 derived colonospheres at rutin IC_30_ and IC_50_ concentrations. Experiments were performed in triplicate and represented by mean ± SEM (standard error mean). *** (*p* < 0.001).

Despite recent medical advances, over half of the patients with colorectal cancer have had tumor recurrence, which renders current treatments ineffectual. This is partially explained by the fact that current treatments try to reduce tumor size rather than destroy it. Researchers believe cancer stem cells (CSCs) have inbuilt resistance mechanisms (stemness/self-renewal and tumorigenic potential) that contribute to disease progression and relapse. Combining CSC targeting with current colon cancer medicines may help avoid recurrence. Recently, rutin, in combination with podophyllotoxin showed protection against radiation-induced gastrointestinal stem cell injury [20]. To the best of our knowledge, no research has been done on the effects of rutin on cancer stem-like cells. To determine the effect of rutin on sphere formation ability, the colon cancer cells were pre-treated with different concentrations (IC_50_ and IC_30_) of rutin and allowed to form colonospheres as per the methodology discussed in Section 2.10. The IC_50_ and IC_30_ observed in the MTT assay in HCT-116 cells were utilized for all the experiments carried out in HCT-116 derived colonospheres. As shown in Figure 5A, the HCT-116 cells formed the colonospheres in a time-dependent manner and comparatively larger spheroids were formed in a five- day incubation period. The results showed that rutin treatment significantly reduced the sphere formation potential in HCT-116 cells in a concentration-dependent manner. Interestingly, at the IC_50_ concentration the spheroid formation potential in the HCT-116 cells was highly reduced. In comparison to bulk tumor cells, colonospheres have a 10–30-fold greater expression of notch signaling, resulting in unrivaled proliferation capability, stemness/self-renewal maintenance, and chemoresistance [63]. In our study, we utilized the *Notch-1* siRNA to silence the *Notch-1* expression in HCT-116 cells and therefore, enabled the formation of colonospheres. The results showed that *Notch-1* siRNA effectively decreased the colonosphere formation potential in HCT-116 cells compared to the scrambled (non-targeted) siRNA (used as negative control) (Figure 5A). Rutin treatment and siRNA mediated *Notch-1* silencing experiments indicated that the notch signaling inhibition mediated the reduced sphere formation potential in colon cancer cells. We also studied whether rutin could down-regulate the notch signaling pathway by inhibiting gamma secretase in the colonospheres. For this, we studied the level of the GS-mediated notch receptor cleaved product (NICD) formation in rutin treated colonospheres. The effect of the rutin on the expression profile of the notch target genes was also studied. At the test concentration, rutin treatment significantly decreased the notch target gene *viz.*, *Hes-1* and *Hey-1* at mRNA levels (Figure 5B). Similar results were obtained in *Notch-1* siRNA treated colonospheres (Figure 5C). Rutin treatment in colonospheres significantly decreased the NICD and the *Hes-1* protein level in a concentration-dependent manner (Figure 5D). We also studied the effect of rutin treatment on stemness/self-renewal markers in colonospheres. The results showed that rutin (at IC_50_ and IC_30_ concentrations) and the *Notch-1* siRNA treatment significantly reduced the mRNA expression of stemness/self-renewal markers (*Nanog*, *Sox2*, *CD44*, and *c-Myc*) in the HCT-116 derived colonospheres (Figure 5E,F). Furthermore, we found that rutin lowered the *c-Myc* protein expression in the test colonospheres in a concentration-dependent manner (Figure 5G). These results prove that rutin treatment decreases sphere formation potential in HCT-116 cells by inhibiting the GS meditated notch signaling pathway.

## 4. Conclusions

Gamma secretase is an important target to inhibit the notch signaling pathway in cancer and cancer stem-like cells. In the present study we have investigated the gamma secretase-mediated notch-signaling inhibition potential of rutin by using computational biology approaches combined with in vitro validation in HCT-116 colon cancer cells and colonospheres. Rutin showed a structurally and energetically stable binding with the catalytic site of GS in the molecular docking and molecular dynamics simulation study. Furthermore, in in vitro experiments rutin efficiently decreased the activity in the notch promoter and downregulated the expression of notch-responsive genes at mRNA level in colon cancer cells. Moreover, rutin treatment inhibited the notch signaling pathway and decreased the spheroid formation potential in colon cancer cells. Overall, this study proposes rutin as a promising natural gamma secretase-mediated notch-signaling inhibitor which could be studied in pre-clinical and clinical setups alone or in combination with other drugs.

## Data Availability

Not applicable.

## Supplementary Materials

The following supporting information can be downloaded at: https://www.mdpi.com/article/10.3390/metabo12100926/s1, Table S1: Average value of analysed MD parameters with standard deviation; Figure S1: System quality checks parameters of rutin-gamma secretase complex, DAPT-gamma secretase and unbound gamma secretase catalytic subunit, Figure S2: Effect of Notch1 siRNA transfection of expression of activated notch 1 (NICD) in HCT-116 cancer cells in 48 h and 72 h treatment.
