# Supplementary material for: Rutin Potentially Binds the Gamma Secretase Catalytic Site, Down Regulates the Notch Signaling Pathway and Reduces Sphere Formation in Colonospheres
Source: Metabolites. 2022 Sep 29;12(10):926. doi: 10.3390/metabo12100926 (PMC9610901; doi:10.3390/metabo12100926)
Supplement: Supplementary file 1 [file metabolites-12-00926-s001.zip › metabolites-1905607-supplementary.pdf]

Supplementary Table S1: Average value of analysed MD parameters with standard deviation

**Parameter**	**Unbound GS**	**DAPT bound GS**	**Rutin bound GS**
RMSD	0.2169566 ± 0.02246795	0.2088716 ± 0.02172804	0.1613630 ± 0.01378015
RMSF	0.1206085 ± 0.06132240	0.1060164 ± 0.05394654	0.1134249 ± 0.04999034
Rg	2.062944 ± 0.01101486	2.1013790 ± 0.00672838	2.0883431 ± 0.00679076
SASA	176.6353 ± 2.859751	171.4507 ± 2.233960	172.7794 ± 2.339975
Intramolecular H-bond	255.4928 ± 5.793144	262.6181 ± 5.85455	253.0453 ± 5.935822
Protein-solvent H-bond	328.5145 ± 10.65406	305.8860 ± 11.47665	313.1300 ± 10.64456

Supplementary Figure S1:

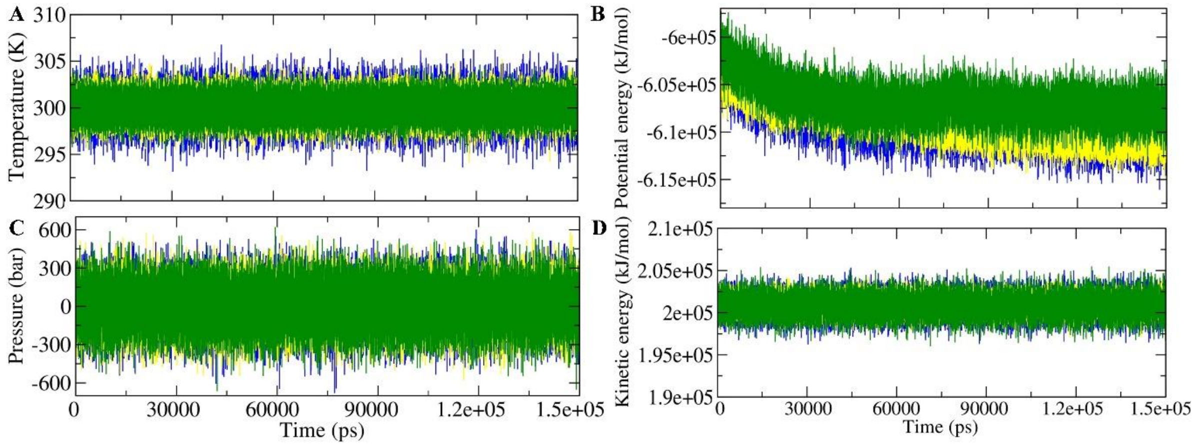

Supplementary figure S1: System quality checks parameters of rutin-gamma secretase complex, DAPT-gamma secretase and unbound gamma secretase catalytic subunit. (A) Temperature (B) Potential energy (C) Pressure (D) Kinetic energy of the all simulated systems throughout 150 ns MD simulation. Unbound GS (blue), DAPT bound GS (Yellow), rutin bound GS (green).

Supplementary Figure S2:

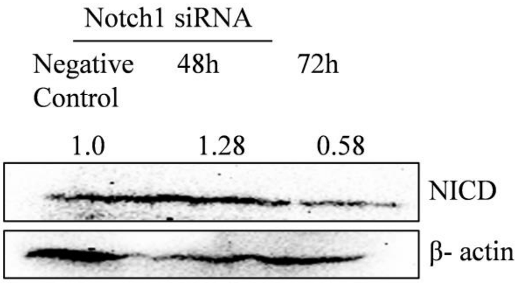

Supplementary figure S2: Effect of Notch1 siRNA transfection of expression of activated notch 1 (NICD) in HCT-116 cancer cells in 48h and 72h treatment. NICD catalogue no: #4147 at the dilution of 1:500X.
